# Dating old hollow trees by applying a multistep tree-ring and radiocarbon procedure to trunk and exposed roots

**DOI:** 10.1016/j.mex.2018.05.015

**Published:** 2018-05-25

**Authors:** Gianluca Piovesan, Franco Biondi, Michele Baliva, Lucio Calcagnile, Gianluca Quarta, Alfredo Di Filippo

**Affiliations:** a DendrologyLab, Department of Agriculture and Forest Sciences (DAFNE), University of Tuscia, Viterbo, Italy; b DendroLab, Department of Natural Resources and Environmental Science, University of Nevada, Reno, USA; c CEDAD (Centre of Applied Physics, Dating and Diagnostics), Department of Mathematics and Physics “Ennio De Girgi”, University of Salento, Lecce, Italy

**Keywords:** Tree-ring and radiocarbon multistep dating of old hollow trees, Hollow tree, Tree dating, AMS, Wiggle matching, Old tree, Radiocarbon, Longevity, Dendrochronology

## Abstract

In the process of dating the oldest trees, which are often hollow, we developed a new method that combines tree-ring cross dating and wiggle matching radiocarbon techniques on wood samples extracted from the stem and from exposed roots. The method can be illustrated by the following steps:

• crossdated tree-ring series from trunk cores reveal a multi-century tree age, and the hollow section is large enough to contain several more years (decades to centuries)
• exposed roots can be cored for acquiring wood samples older than the stem cores and for construction of a floating root average tree-ring series
• if synchronization between stem and exposed roots is unclear, proceed to date the root wood samples by radiocarbon wiggle matching; match root and stem tree-ring series within the radiocarbon-dated period to more accurately date the tree.

This new multistep dating method allowed for refining the age estimation of the oldest Pinus heldreichii tree in Pollino National Park by 166 years, to 789 CE. This tree, which we named *Italus*, was 1229 years old in 2017, making it the oldest, scientifically dated, living tree in Europe. Any study that relies on tree age determination for paleo-reconstructions, for biological and genetic research on what controls longevity, or for understanding structural dynamics and succession in old-growth forests, would potentially benefit from the multistep dating method we tested.

## Graphical abstract

**Figure:**
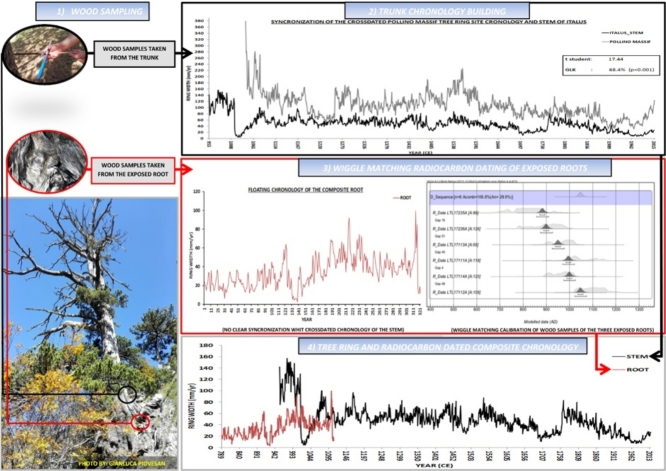

Specifications Table *[please fill in right-hand column of the table below]*

**Table:** 

Subject area	*Select one of the following subject areas:*
	• Agricultural and Biological Sciences
More specific subject area	*Dendrology, Forest biology and ecology, Dendrochronology*
Method name	Tree-ring and radiocarbon multistep dating of old hollow trees
Name and reference of original method	*If applicable, include full bibliographic details of the main reference(s) describing the original method from which the new method was derived.*
Resource availability	*If applicable, include links to resources necessary to reproduce the method (e.g. data, software, hardware, reagent)*

## Method details

### Overview

Measuring the maximum lifespan of old trees and their associated growth patterns is necessary to scientifically assess forest health and dynamics, which is required for long-term conservation of threatened species that survive in rare habitats [1]. However, heart rot is frequent in old trees, especially if deciduous [2], and for this reason wood increment cores underestimate tree age. In particular, the calculation of missing years in the hollow section of the trunk can be biased by the non-linearity of growth processes, so that using the average increment in the innermost visible part of wood cores does not provide reliable estimates. For this reason, in the study of maximum tree longevity, estimated ages of samples without pith are generally excluded from the analyses [3]. However, when tree-ring analyses of trunk cores reveal a multi-century tree age, and the hollow section is large enough to contain several more years (decades to centuries) producing a remarkable estimated age of old hollow trees, our new combined tree-ring and radiocarbon multistep method can be used to improve the accuracy of age estimates. We applied this new procedure to date an old hollow pine (*Pinus heldreichii* H.Christ) named *Italus* that was recently discovered in the Pollino National Park [4].

### Procedure

1 Crossdated tree-ring series from wood cores [5] extracted from the main hollow trunk reveal a noteworthy age for the species and/or for the taxon (e.g. subgenus, genus, family).
2 Missing years belonging to the hollow section of the trunk are estimated using the average growth of the first 20–50 years of the stem cores [6]. If this estimate of the “missing” innermost section is remarkable (e.g. several decades or centuries), determine if large old roots close to the tap root can be sampled (e.g. by coring large exposed roots).
3 Extract wood cores from the innermost part of the exposed root system.
4 Construct a mean floating tree-ring series for the exposed root systems.
5 If synchronization is unclear between root and trunk tree-rings [[7], [8], [9]], then use radiocarbon dating [10].
6 Radiocarbon date at least 2 samples for each root cores at a distance > 20–30 years for wiggle matching analyses [11].
7 Wiggle matching analysis is based on the floating average root tree-ring series.
8 Search within the radiocarbon-dated period a significant tree-ring synchronization to construct a composite stem and root tree-ring series.

### Method validation

This multistep tree-ring and radiocarbon dating procedure for age determination of old hollow trees was applied to a *Pinus heldreichii* named *Italus*, located in Pollino National Park, southern Italy [4].

**Fig. 1:**
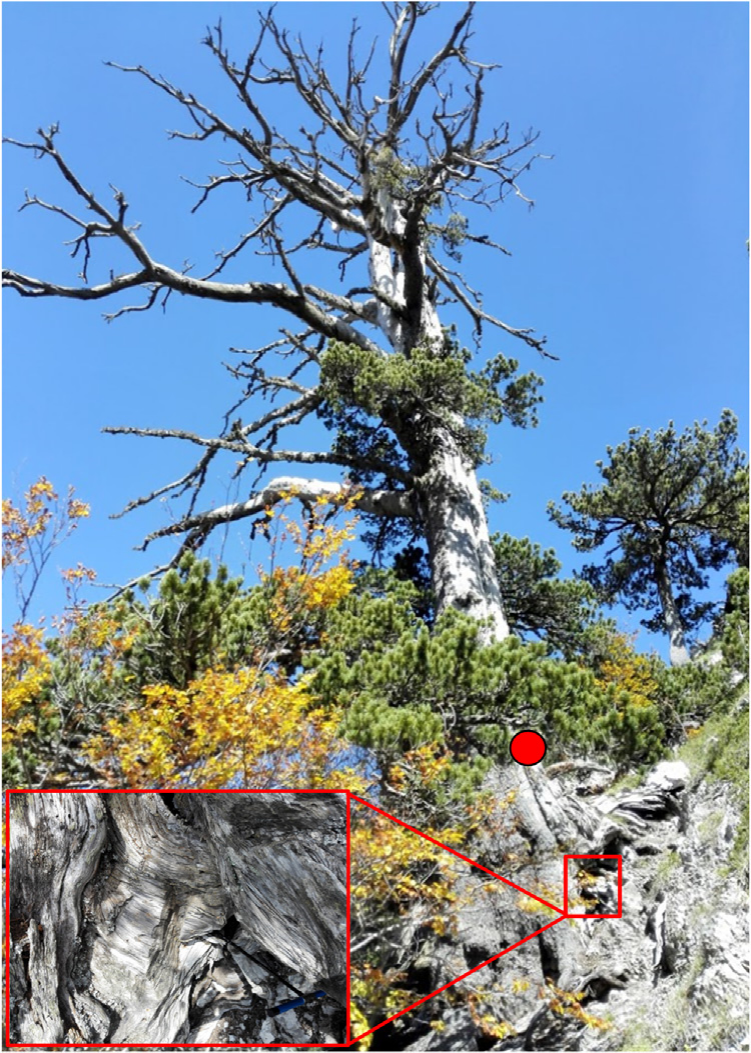
The millennium-old pine tree named *Italus*. Wood cores were extracted at breast height (∼1.3 m; red dot; stem) and from the exposed vertical root system using a modified increment borer (also shown, red square and lower inset box).

**Fig. 2:**
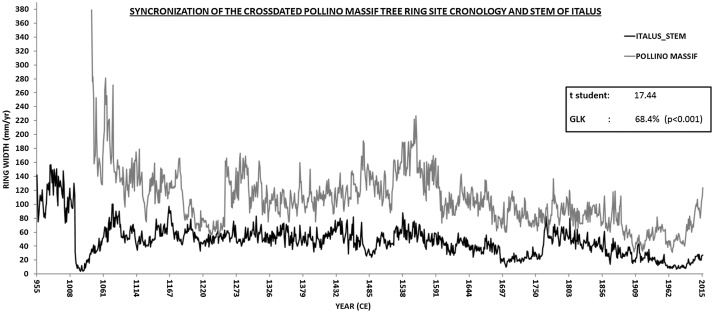
Comparison of crossdated tree-ring series for *Italus* - developed by averaging 4 stem cores - with the Pollino master chronology. Student’s t and Gleichläufigkeit (GLK) statistics, calculated by software Catras [7] are shown in the box. GLK measures the percentage of common signs of year-to-year growth change between two series.
2) Age estimation based on the average width of the innermost 20–50 rings suggested 205–227 rings between the missing pith and the first dated ring. The estimated pith date was 727–749 CE (age range 1290–1268 years), making *Italus* a likely candidate for one of the most longeve trees in Europe.
3) Wood cores were extracted from the exposed root system in two successive periods. During a first campaign, three cores located in the external part of the old exposed root system were sampled (Fig. 3, red box) and used for constructing a floating tree-ring series and for radiocarbon analyses. A second sampling effort (2 wood cores) focused on the internal part of the exposed root system (Fig. 3, yellow box) because of the interesting radiocarbon dating of the first specimens.

1) Four wood cores 5-mm in diameter were extracted from the stem of *Italus* at different height level (0.5–1.5 m) using an increment borer (Fig. 1). A crossdated tree-ring series (Fig. 2) revealed a remarkable age for the species [4,12] and for the subgenus *Diploxylon* ([13], see also Oldlist http://www.rmtrr.org/oldlist.htm).

**Fig. 3:**
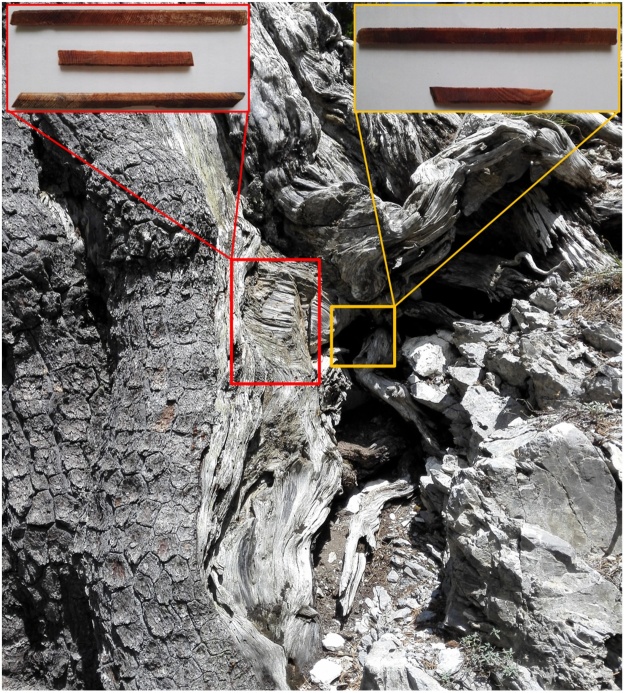
Exposed root sampling: on the left (red box) external root cores used for radiocarbon analysis, and on the right (yellow box) internal root cores sampled in a second phase of the study using a modified increment borer. Note that the sampled roots are located well below the root collar (see also Fig. 1). Moreover the coring was performed in a section of the exposed vertical root system that lacked the bark and a large portion of outer rings.
4) Single floating root series were 117–322 years long, and well-synchronized with one another (Fig. 4a,). A composite root series of 322 years was constructed by merging all root samples (Fig. 4b).

**Fig. 4:**
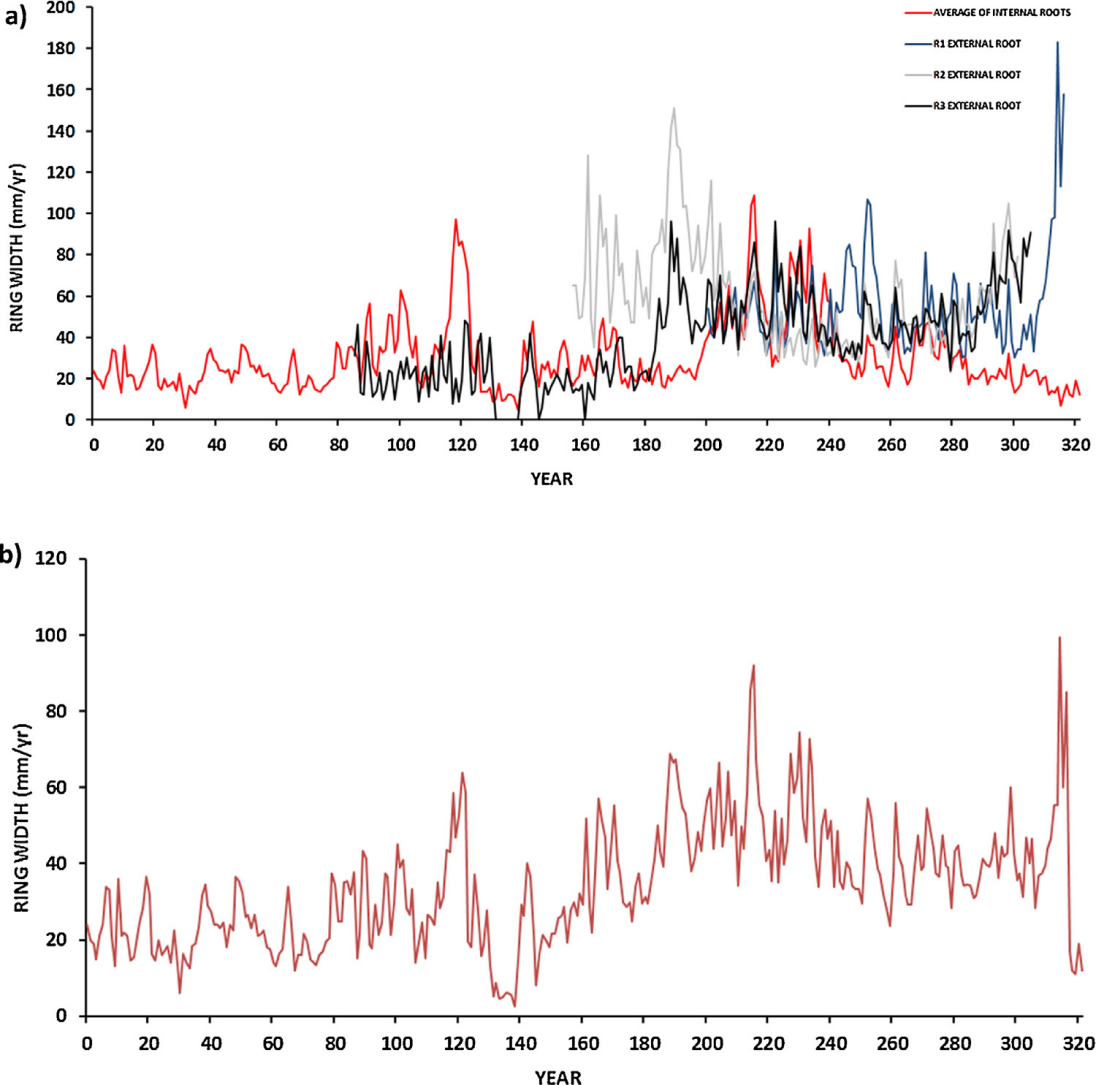
a) Floating ring-width series of the external and internal exposed root system. All series are synchronized at a highly significant level (see also Appendix S1 of [5]). b) Floating average root tree-ring series were constructed by merging all core samples from the exposed root system.
5) No clear crossdating emerged between the stem and the exposed roots. This is not a surprising result because ring irregularities are a frequent character of root growth, hence cross-dating is more difficult [9].
6) Six wood samples from the 3 exposed external root cores (two for each core, Fig. 3, see also [4] for details) were chemically processed in order to extract the purest cellulose form by following the “BABAB” method reported in [14]. The purified sample material was combusted to CO_2_ in sealed quartz tubes, cryogenically purified and catalytically reduced to graphite by using H2 as reducing agent [15]. The ^14^C content was then measured with the AMS system installed at CEDAD and based on a 3 MV Tandetron Accelerator [16].
7) Calibrated radiocarbon ages of six wood samples (Fig. 5), two for each root core extracted during the first sampling campaign, were calibrated using Bayesian statistical tools in the software OxCal [11]. Every couple of samples from the same root (Root1, Root2, Root3) was calibrated independently from the others (Fig. 6)

**Fig. 5:**
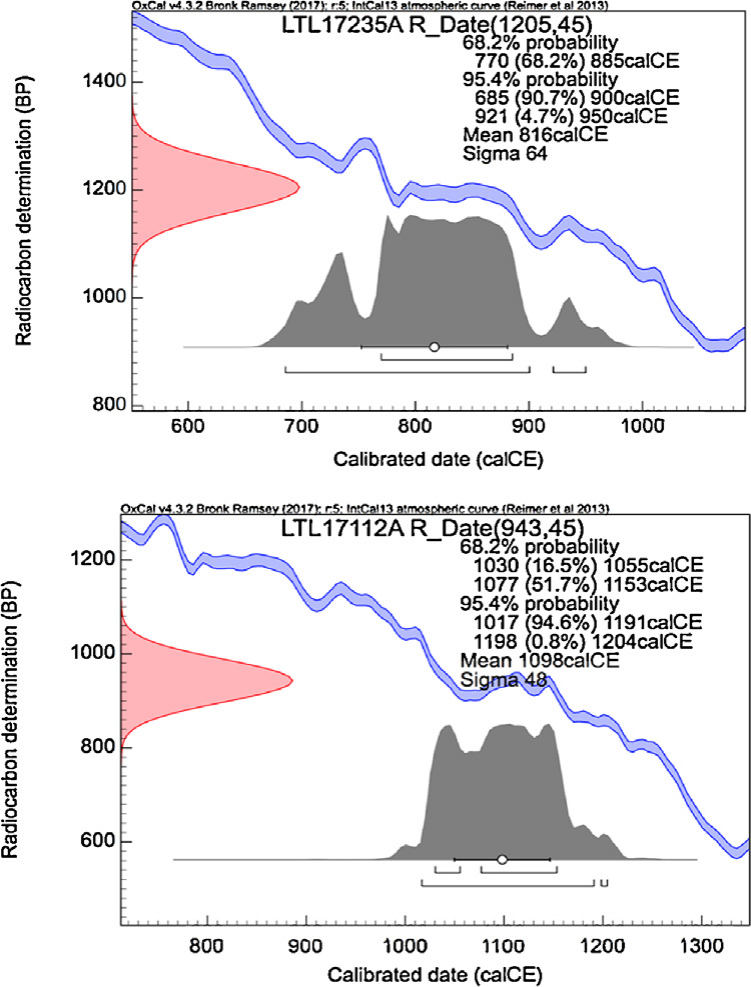
Calibration of the conventional radiocarbon date assigned to the oldest (LTL17235 A, top graph) and the youngest (LTL17112 A, bottom graph) wood samples from the external exposed roots.

**Fig. 6:**
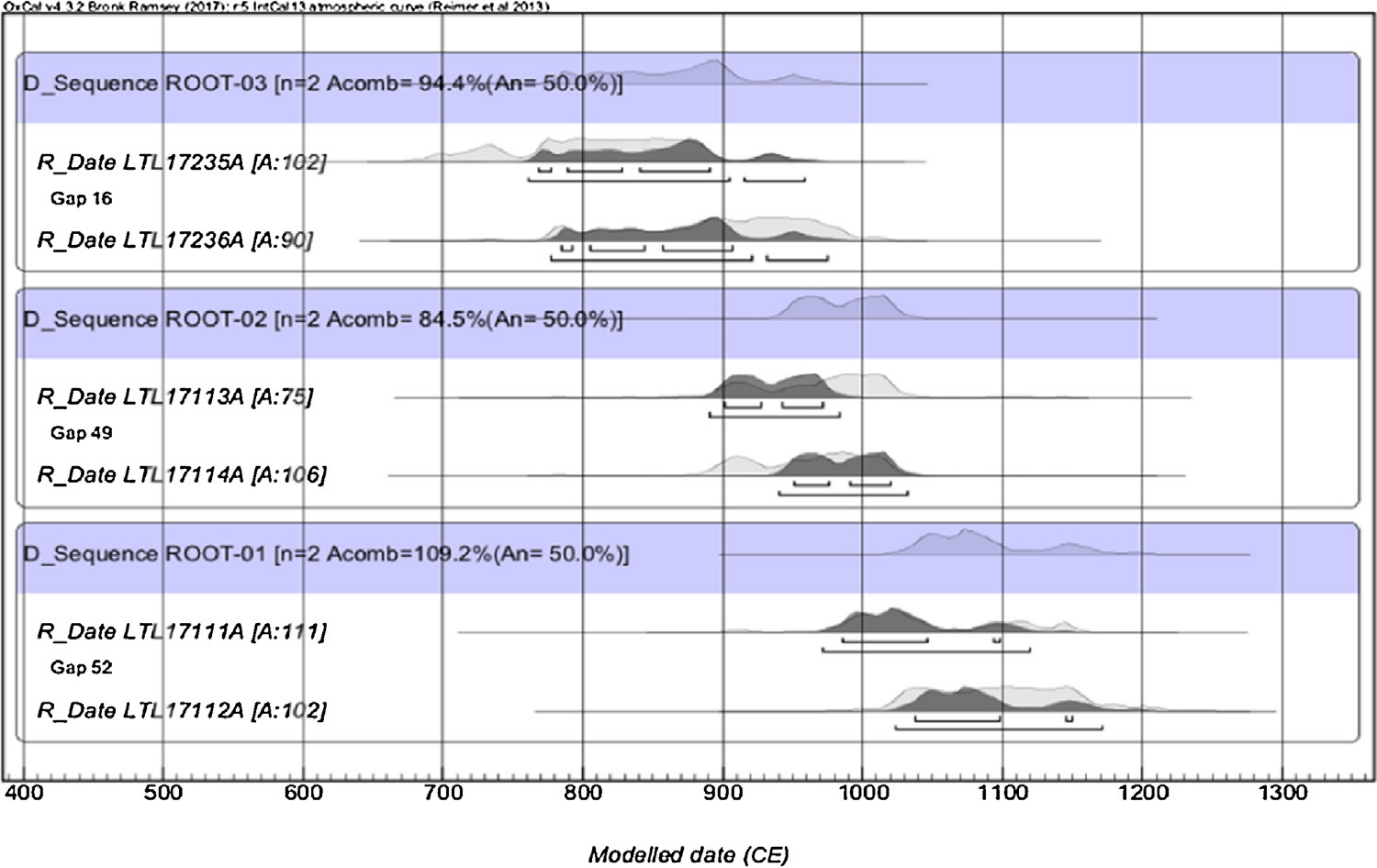
Wiggle matching calibration of wood samples from the three external exposed roots. Every couple of samples from the same root (Root1, Root2, Root3) was calibrated independently.
8) Wiggle matching calibration of wood samples of the three exposed roots was based on the floating tree-ring series (Fig. 7).

**Fig. 7:**
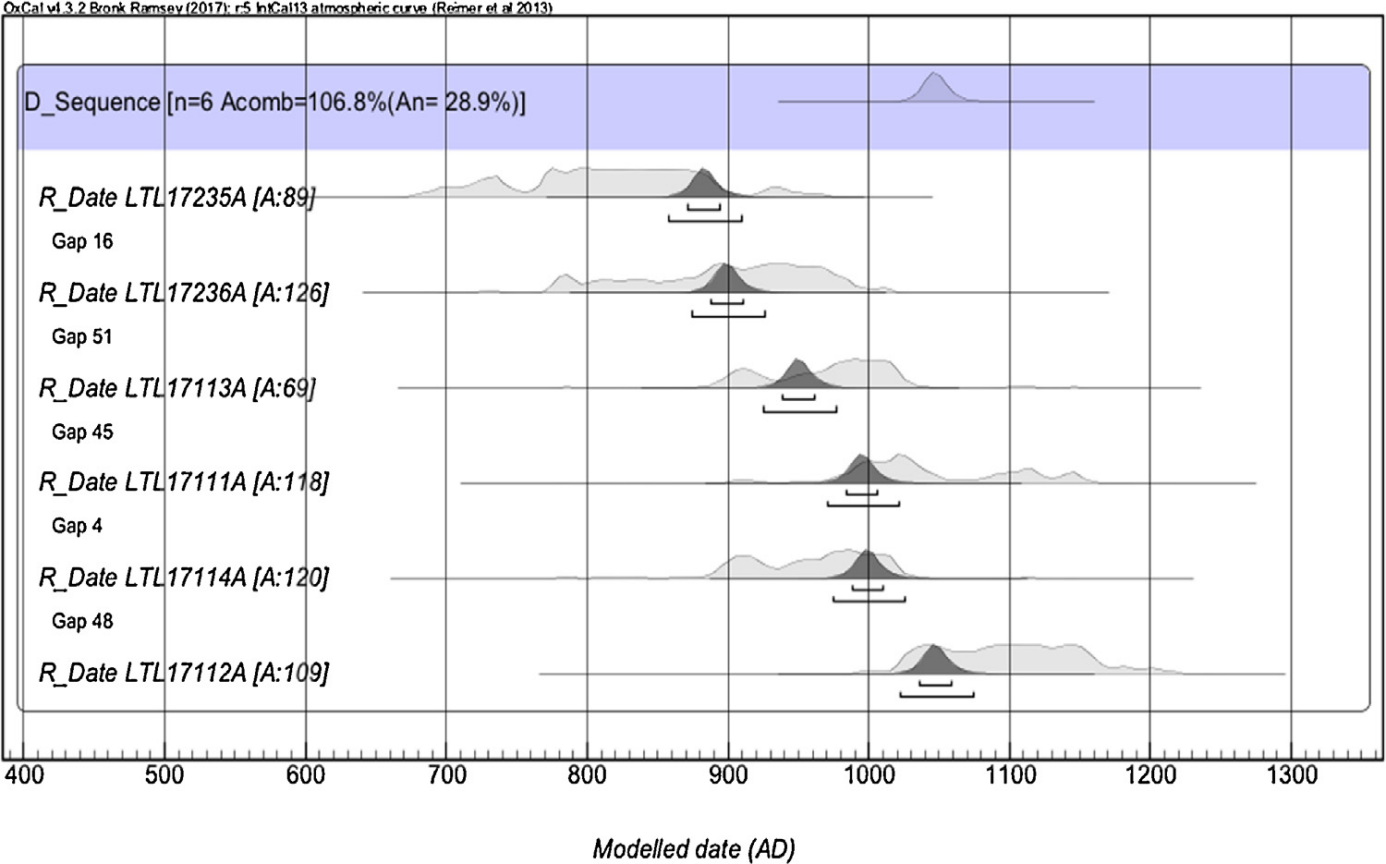
Wiggle matching calibration of wood samples from the three external exposed roots based on the floating tree-ring series developed for the root system. The three exposed roots were considered as a whole, i.e. considering the time separation among tree-ring series as determined by crossdating.
9) Within the dating window provided by wiggle matching, one of the external root tree-ring series (R2) was matched to the stem series (*t-*values of 3.45 and GLK of 63.0%, significant at 99%) assigning year 878 CE to the oldest radiocarbon-dated sample, well within the radiocarbon dated interval (Fig. 8).

**Fig. 8:**
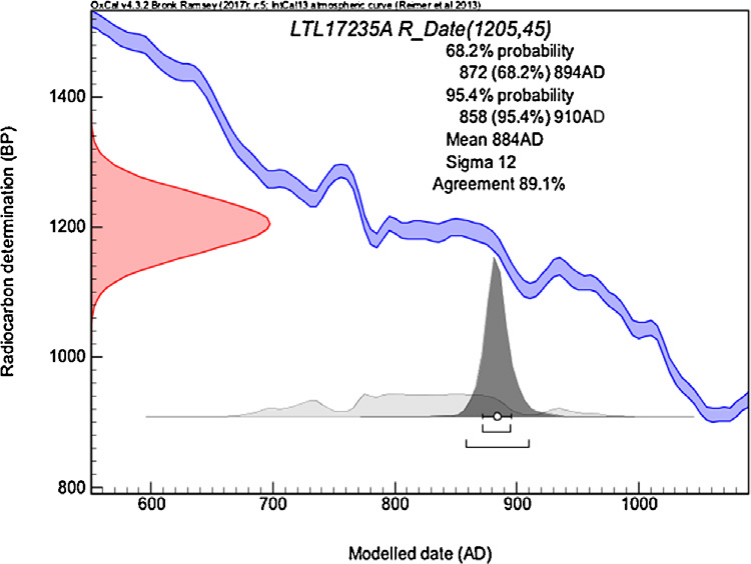
Radiocarbon dating of the oldest tree-ring sample from the exposed roots, which was placed at 884 ± 12 CE by wiggle matching. The sample belongs to the external exposed root R3.
10) The ring-width series from the stem combined with the radiocarbon-dated tree-ring root series of *Italus* (Fig. 9) gave a minimum age of 1229 years, making *Italus* the oldest dated tree in Europe [4,12].

**Fig. 9:**
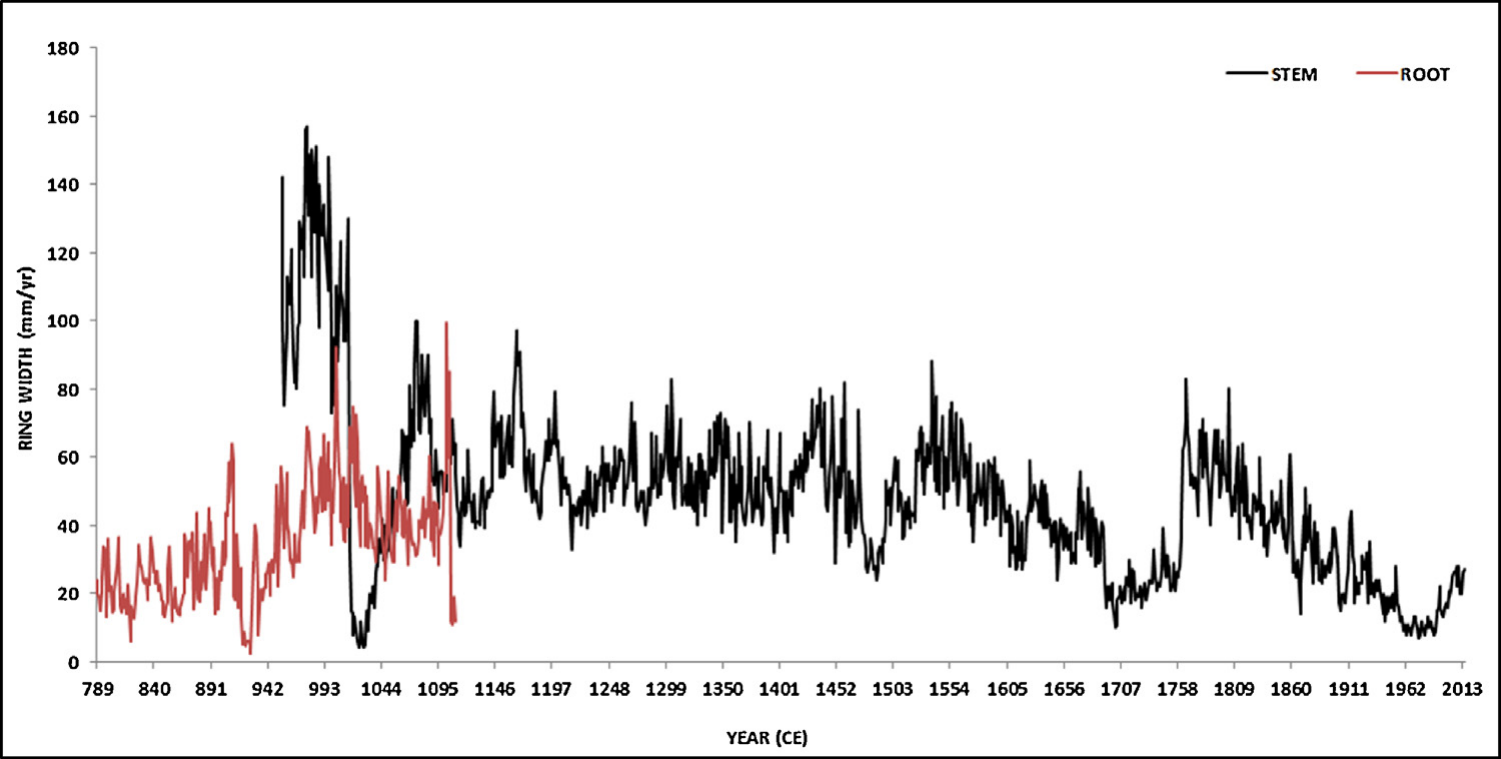
Tree-ring series of *Italus*: crossdated ring-width series from the stem (black) were matched with the radiocarbon-dated average tree-ring root series (red).

### Method limitation

This method could be applied to trees that develop exposed, large, and long lived roots, as in the case of most *Pinus* species.
